# Successful generalization of conceptual knowledge after training to remember specific events

**DOI:** 10.3389/fcogn.2024.1324678

**Published:** 2024-09-26

**Authors:** Troy M. Houser, Anthony Resnick, Dagmar Zeithamova

**Affiliations:** ^1^Department of Psychology, University of Oregon, Eugene, OR, United States; ^2^Institute of Neuroscience, University of Oregon, Eugene, OR, United States; ^3^Annenberg School for Communication, University of Pennsylvania, Philadelphia, PA, United States

**Keywords:** category learning, prototype theory, exemplar theory, paired-associate learning, incidental learning, generalization (psychology)

## Abstract

**Introduction:**

Categorization involves grouping information to make inferences and support novel decisions. In the laboratory, category learning tasks commonly involve trial-and-error where participants are instructed to classify stimuli and learn through feedback. Here, we tested across two experiments whether people can acquire category knowledge in an incidental manner by associating category members with other information that itself is structured, and how it compares to acquiring category knowledge directly through feedback-based classification training.

**Methods:**

Subjects were trained to remember specific associations consisting of cartoon animals paired with animal-specific background scenes. Animals presented on forest vs. mountain scenes were members of two prototype-based categories, but this was not conveyed to the participants. Spontaneous category learning was tested by asking participants to guess habitat (mountains, forests) for old and new cartoon animals without feedback.

**Results:**

We found that participants spontaneously acquired category knowledge, showing high categorization accuracy for new animals, comparable to a group that underwent a traditional feedback-based classification training with the same stimuli. Strategy analysis showed that the majority of participants in both groups abstracted the central tendency of the categories, albeit a somewhat larger proportion of subjects relied on memory for specific training exemplars after paired-associate learning. Partial evidence was found for the hypothesis that generalized knowledge emerged at the expense of memory for specific animal-scene associations.

**Discussion:**

The findings show that despite the goal to remember specific information that required differentiation of stimuli within categories, subjects can spontaneously acquire category knowledge, generalizable to novel stimuli in a way comparable to traditional supervised classification training. This work provides new insights into how category learning can proceed under more naturalistic demands.

## Introduction

Categorization is important for many facets of cognition, including decision-making, object recognition, and language processing. The study of categorization has primarily utilized supervised category learning tasks that consist of showing participants a stimulus and teaching them its category membership through corrective feedback (Love, 2002; Nosofsky et al., 1994; Shepard et al., 1961). The ability to generalize category labels to new stimuli is then taken as evidence for the formation of category knowledge. This work has led to the development of several successful categorization models, including exemplar models that emphasize the role of memory for specific category exemplars (Medin and Schaffer, 1978; Nosofsky, 1986) and prototype models that emphasize the formation of abstract category representations (Posner and Keele, 1968; Smith and Minda, 1998). While we have learned much from this traditional approach, overt category decisions coupled with explicit feedback likely do not capture the range of ways in which we acquire category knowledge in the real world.

Importantly, category knowledge can also be acquired *incidentally*, without explicit instruction. For example, imagine trying an assortment of dishes from a foreign country. Perhaps, the dishes have similar spices, aromas, and flavors. Then, your friend tells you that the dishes are part of Thai cuisine. In the future, you will likely be able to differentiate Thai from other cuisines, indicating that, while you may not have known what type of food you were eating, your brain was implicitly grouping similar sensory information together to form a unique category.

Incidental perceptual category learning studies in the auditory (Gabay et al., 2015, 2023; Lim et al., 2019; Roark et al., 2022) and visual (Aizenstein et al., 2000; Bozoki et al., 2006; Kéri et al., 2001; Love, 2002; Reber et al., 2003; Wattenmaker, 1993) domains have demonstrated how category structure can be extracted in the absence of explicit instruction. In a typical incidental perceptual categorization task, participants are shown a series of stimuli and then informed that all of those stimuli form a category. They are then asked to indicate for a series of new stimuli whether or not each stimulus belongs to the same category (referred to as an A/not A task; e.g., Ashby and Maddox, 2011; Zeithamova et al., 2008). Less frequent has been work on unsupervised acquisition of category structures containing two or more categories (Ashby et al., 1999; Love, 2003). While learning in these paradigms is unsupervised, lacking feedback, the aim to discover the underlying category structure is typically explicitly instructed and its success is variable, influenced for example by the order in which the stimuli are presented (Clapper, 2006; Clapper and Bower, 1994, 2002; Zeithamova and Maddox, 2009).

More recently, a question arose whether it is possible to incidentally learn categories when explicitly focused on individuating each stimulus and when categories are not clearly dictated by an underlying similarity structure among stimuli. A recent study utilized the presence of a shared label (family name) as one way to provide an opportunity to spontaneously form category knowledge (Ashby et al., 2020). Participants learned a set of paired associates, each consisting of a face and their full names (e.g., “Peter Miller”). To generate a similarity structure among the faces, face stimuli were created by morphing together two never-studied “parent” faces. Some of the faces with a shared parent were assigned a shared family name while other faces with a shared parent were assigned different family names, enabling to tease apart the effects of physical similarity and category membership. After learning, participants demonstrated a category bias in perceptual similarity ratings such that faces with shared family names were rated as more similar than faces that were equated for physical similarity but had different family names. Participants were also highly successful in generalizing family names to never-studied faces, with the degree of generalization being predicted by the emerged perceptual biases. This finding was later replicated in a separate fMRI scanned sample, which provided evidence for category representations forming spontaneously in the brain while participants were still learning the unique full names for each face (Ashby and Zeithamova, 2022). The nature of those category representations, such as whether they tend to be exemplar-based or generalized, is however not straightforward to adjudicate for such stimuli.

**Figure 1:**
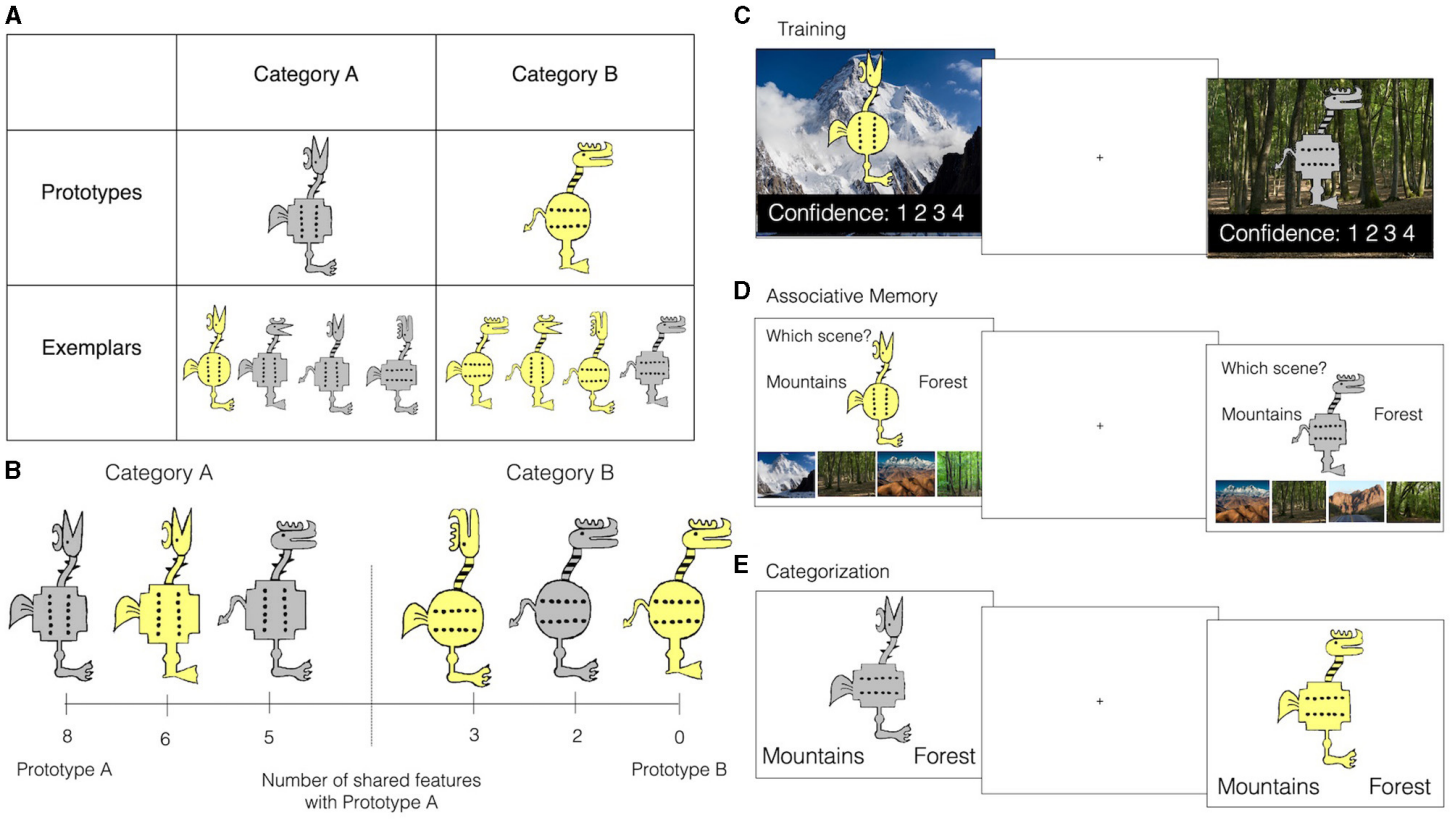
Experiment design. **(A)** Example stimulus set and the corresponding prototypes. Top row: category prototypes. Bottom row: example training set, divided into their correct categories. **(B)** Stimuli were cartoon animals varying along 8 binary dimensions (features). Two maximally distinct stimuli (different on all 8 dimensions) served as category prototypes. Each stimulus can be characterized by the number of features by which it differs from each prototype (referred to as physical distance). **(C)** Example training trial. Background and response options appeared first. The stimulus took 2 s to gradually appear, at which point subject can report how confident they are they will remember the animal-scene association. Fixation screen immediately follows response. **(D)** Example associative memory test trial. Each training stimulus was shown with 6 possible response options: Correct scene, lure scene from the same category, two foil scenes from the other category, and two verbal category labels (“Mountains”, “Forests”) if a participant remembers the scene category but not the exact scene. **(E)** Example category generalization test trial. Participants were asked to guess the correct scene category for a set of training and new (generalization) animals.

Here, we build upon and extend this work in two directions. First, we tested whether people spontaneously acquire category knowledge even when no shared label is provided, through a mere association of each stimulus with other information that itself has category structure. Second, we utilized binary-dimension stimuli, a type of stimuli used extensively in prior categorization studies and suitable for the use of formal categorization models that can estimate underlying category representations from the patterns of participants' responses. Participants were asked to remember a set of paired associates, consisting of a cartoon animal and its preferred habitat (a background scene unique to each animal). Though participants needed to remember the specific scene for each cartoon animal, the scenes fell into two categories, mountains and forests, providing the opportunity for the participants to spontaneously organize the cartoon animals in line with their associated scene type. After paired-associates training, we measured category knowledge by testing people's ability to generalize category membership (forest or mountain habitat) to novel animals. Further, the binary dimension stimuli in a form of cartoon animals allowed us to utilize two well-established categorization models to determine the type of representations people rely on after they acquired category information incidentally. The *exemplar model* posits that people store individual instances in memory (Figure 1A) and categorize new instances based on their similarity to all stored exemplars (Medin and Schaffer, 1978; Nosofsky, 1986). By contrast, the *prototype model* posits that people extract the central tendency across category exemplars (Figure 1A) which subsequently guides generalization behavior (Posner and Keele, 1968; Smith and Minda, 1998). We hypothesized that people trained to remember specific associations will successfully form and generalize category knowledge, but may increasingly rely on memory for specific exemplars rather than abstracted central tendencies (prototypes). To test this hypothesis, we utilized a category structure that leads to predominantly prototype representations when learned through traditional feedback-based classification training (Bowman and Zeithamova, 2018), and assessed (1) people's ability to incidentally acquire and subsequently generalize category knowledge, and (2) the impact that incidental learning has on generalization strategy. Finally, we explored whether the type of category representations that people formed (prototype or exemplar) related to how well they remember specific animal-scene associations requiring them to individuate category members, given the theoretical proposals suggesting a trade-off between memory specificity and generalization (McClelland and Goddard, 1996; Shohamy and Wagner, 2008; Varga et al., 2019). Overall, this work moves us closer to understanding the nature of category learning under more naturalistic demands.

## Experiment 1

### Method

#### Participants

Healthy participants (*n* = 71; 50 females) were undergraduates recruited from the University of Oregon via the university's SONA research system. Ages ranged from 18 to 30 (M = 19) years. Participants received course credit for their participation. All participants provided written informed consent and experimental procedures were approved by Research Compliance Services at the University of Oregon.

#### Materials

##### Stimuli

The complete stimulus set is freely available through the Open Science Framework (http://osf.io/8bph2). Stimuli consisted of cartoon animals that varied along 8 binary feature dimensions: color (yellow/gray), shape of feet (clawed/webbed), shape of body (squared/circular), shape of tail (devil tail/feather tail), dot orientation on body (vertical/horizontal), pattern on neck (stripes/horns), head shape (with beak/with horn), and head orientation (forward/up). Two stimuli with the maximally different number of features were the prototypes for Category A and Category B for all participants (Figure 1A). Physical similarity between stimuli was defined as the number of shared features (Figure 1B). The inverse, physical distance, was defined as the number of differing features. Category A stimuli shared more features with prototype A than prototype B and vice versa. Stimuli that were equidistant from both prototypes were not used.

##### Training set

Four training sets were created and assigned to participants randomly. All training sets included four stimuli per category (eight training stimuli in total), each differing from their respective category prototype by two features. An example training set with the corresponding prototypes is presented in Figure 1A. Prototypes themselves were never presented during training. Because the majority of the training exemplars differed from each other by four features, whether they belonged to the same or opposing category, the similarity structure among stimuli did not fully dictate category membership. Instead, the membership was determined via the associated scenes.

##### Training scenes

Each training animal was paired with an animal-unique scene (8 scenes total) for the paired-associate learning task (Figure 1C). The scenes were distinctive from each other, but fell into two categories, mountains and forests. Training animals that belonged to one category were all paired with scenes from one scene type, training animals that belong to the other category were all paired with scenes from the other scene type. In total, there were eight unique animal-scene paired-associates used during training.

##### Testing set

Four testing sets were created and assigned to participants based on which training set they were administered. All testing sets included the 8 training stimuli (each presented twice) and 50 novel stimuli. Novel stimuli consisted of 8 randomly selected animals at each distance from the category prototypes (e.g., eight new animals one feature different from prototype A, eight new animals two features different from prototype A, and so on), excluding equidistant stimuli. The test sets also included the two previously unseen prototypes, each shown twice. Repeating the training stimuli and prototypes helps ensure dissociable prototype and exemplar model predictions (Bowman and Zeithamova, 2018; Kéri et al., 2001; Smith et al., 2008).

#### Experimental design

##### Training

Participants first completed a training session where they were instructed to remember which background scene is the habitat for each unique training cartoon animal. Importantly, while each of the eight background scenes was unique, all scenes fell into two categories: mountains or forests, with the habitat type aligning with category structure. In other words, all four category A exemplars were paired with one of the four unique forest scenes while all four category B exemplars were paired with one of the four unique mountain scenes. Participants were not informed about the existence of the scene or animal categories and instead the task resembled a paired-associate learning task as used in episodic memory studies.

On each trial, participants were first shown one of eight background scenes that filled the screen. The associated cartoon animal then took 2 s to materialize by gradually increasing the image's opacity. Participants were then prompted to report how confident they are that they would remember the scene habitat for the particular cartoon animal using a button press corresponding to “1 = Definitely Forget”, “2 = Maybe Forget”, “3 = Maybe Remember”, or “4 = Definitely Remember”. Participants had 5 s to make a decision but the trial advanced if a response was registered sooner. An example training trial is shown in Figure 1C. Participants completed five blocks of training, each containing five repetitions of all training items with self-paced breaks between the blocks. The total number of training trials was two hundred (8 animal-scene pairs X 5 repetitions per block X 5 blocks = 200 trials). Stimulus order was randomized within each block.

##### Associative memory test

Following training, participants were administered an associative memory test, where they were shown each training stimulus along with four scene options and instructed to respond which scene they think the stimulus was paired with during training (Figure 1D). Scene options always consisted of two mountain and two forest scenes: the correct scene, a lure of the same type, and two foils from the other habitat type. That way, the choices provided did not offer any cues with respect to which scene type (forest or mountain) is correct. Participants were also given the option of responding “Mountains” or “Forests” if they could not recall the specific scene.

##### Category generalization test

As the final portion of the session, participants were administered a surprise generalization test. On each trial, they were shown a training or a new (generalization) cartoon animal and instructed to respond either “Mountains” or “Forest” via button press (Figure 1E). The categorization test consisted of one block of 68 trials with 5 s stimulus presentation and 7 s fixation. Trials advanced earlier if responses were registered before 5 s. There was no feedback during the generalization test.

#### Statistical analysis

Raw data and the code necessary to reproduce the plots for Experiment 1 can be found at https://osf.io/dhmks.

##### Prospective memory judgments during training

Prospective memory judgements at training were mainly collected to ensure continued attention during observational learning and were not of primary interest. Nevertheless, we conducted a repeated-measures ANOVA to see if participants' confidence in their memory for the scene-animal associations increased over time. For each participant and each block, we computed the mean confidence rating. We expected to see an effect of training block. Only 49 participants were included in the prospective judgments analysis: due to technical error, data from the final training block was not saved for 21 participants and one participant made no responses for the first training block.

##### Associative memory

To evaluate how well participants remembered the specific animal-scene associations, we calculated the proportion of correct scene responses during the associative memory test for each participant. For completeness, we also computed and report the proportion of responses to all other unique options (i.e., to lure scene, specific foil scenes, and correct and incorrect general category labels).

##### Categorization accuracy during generalization test

To estimate how well participants generalized incidentally learned category information, we calculated the average accuracy of responses for each participant and compared these averages to chance level (50%) using a single sample *t*-test. Next, we tested to what degree generalization responses are modulated by the similarity to prototypes and whether the data exhibits a similarity gradient as commonly reported in category learning studies. For each participant, we first calculated the mean categorization accuracy across all generalization (new) trials at each distance from its prototype. These scores were submitted to a repeated measures ANOVA to test for an effect of distance on generalization performance. Additionally, we compared categorization accuracy for old (training) animals and new (generalization) animals at the same distance from the prototypes (two features difference, as all training animals varied from their respective prototypes by two features) using a paired *t*-test. An old-advantage, or greater accuracy for training (old) than generalization (new) stimuli at the same distance, indicates a contribution of the memory for specific instances to categorization and is often observed even when behavior is otherwise dominated by a prototype strategy (Bowman and Zeithamova, 2018). Given that we instructed participants to remember specific events, we predicted that old animals would be categorized more accurately than new animals.

##### Categorization strategies during generalization test

To identify the generalization strategies that people used, we fit prototype and exemplar models to the trial-by-trial responses from the generalization phase for each participant. Prototype models assume that category structures are represented by their prototype, and thus, they compute the similarity of each generalization stimulus to each prototype. Perceptual similarity is modeled as an exponential decay function of physical similarity (Shepard, 1958, 1987), while taking into account differences in attention to specific features. Formally, the similarity *S* of a stimulus *x* to category A is:


$$\begin{array}{l} S_{A}\left(x\right)=\exp\left[-c\sum {\left(w|x-proto_{A}{|}^{r}\right)}^{\frac{1}{r}}\right], \end{array}$$

where *c* is a sensitivity parameter that controls the rate of decay (estimated from the data), *w* is a vector of attention weights with length equal to the number of stimulus features (estimated from the data and constrained to sum to one), and *r* is the distance metric (set to 1 to reflect city-block distance metric typically used for binary features). In total, there are nine participant-specific parameters estimated from their pattern of responses: one c parameter and eight attention weights, one for each stimulus dimension. Thus, the models can account for intra-individual and inter-individual variability in feature saliency.

Exemplar models assume that categories are represented by their exemplars, or individual instances. These models calculate similarity between each stimulus and a category by computing the summed similarity of the stimulus and all the training stimuli from the given category (Nosofsky, 1987):


$$\begin{array}{l} S_{A}\left(x\right)=\underset{y\in A}{\sum }\exp\left[-c\sum {\left(w|x-y{|}^{r}\right)}^{\frac{1}{r}}\right]. \end{array}$$

Here, *y* represents a training stimulus from category A and all other parameters are the same as in the prototype model.

To transform similarity scores to probabilities, we used a Luce choice rule (Luce, 1963):


$$\begin{array}{l} P\left(A|x\right)=\frac{S_{A}\left(x\right)}{S_{A}\left(x\right)+S_{B}\left(x\right)}. \end{array}$$

Using these equations, we obtained best fitting parameters from both models for each participant via maximum likelihood techniques. Specifically, we minimized the negative log likelihood by adjusting parameter estimates with gradient descent. To obtain best fitting parameter estimates, we used the *Rsolnp::solnp* function (Ye, 1987) as implemented in R, which optimizes model fit with general nonlinear optimization augmented with Lagrange multipliers. We used the method of Lagrange multipliers to impose an equality constraint on the attention weight parameters such that they had to sum to 1. We used noninformative priors, set all lower bounds to 0, and the upper bound for the sensitivity parameter was set to 20.

##### Model selection

After optimization, prototype and exemplar model fits were compared at the individual level. A number of studies have demonstrated how group-level trends in generalization can be obtained by superimposing subgroup trends (Ashby et al., 1994; Lee et al., 2018; Lee and Livesey, 2018; Lovibond et al., 2019), highlighting the need for individualized assessment of generalization behavior (Zaman et al., 2023). Model fits were compared to each other and to chance to estimate the best fitting categorization strategy for each individual participant. We used a Monte Carlo simulation approach for model selection, as described previously (Bowman and Zeithamova, 2018; Kroese and Rubinstein, 2012). Each participant was assigned one of four strategy labels: exemplar, prototype, comparable fit, or random. First, we shuffled empirical responses 10,000 times and obtained model fit values for each permutation to generate subject-specific null distributions for both computational models. We then compared empirical model fits against the subject-specific distribution of these null model fits. If the empirically observed fits were better than 95% of the simulated fits, we conclude that the model describes the data better than assuming that the participant responded randomly (p < 0.05, one-tailed). Participants who had neither prototype nor exemplar fit reliably better than chance were given “random” as their strategy label. For the remaining participants, we then directly compared the prototype and exemplar model fits to each other. To test for differences in model fits for each participant, we compared relative model fit differences, [(fit_prototype_ – fit_exemplar_)/(fit_prototype_ + fit_exemplar_)] to the relative model fit differences of simulated fits. One model was deemed a winner (i.e., subject was deemed a “prototypist” or “exemplarist”) if the difference in empirical fits appeared by chance with a frequency of < 25% (*p* < 0.25, two-tailed). This approach has been shown to be more suitable for model comparison than simply comparing raw fits for two reasons: (a) the exemplar model tends to fit even random data slightly better than the prototype model, suggesting that equal number of parameters does not guarantee equal model flexibility, and (b) utilizing a confidence threshold before one model is deemed a winner helps differentiate negligible model fit differences from more meaningful ones (see Bowman et al., 2020; Bowman and Zeithamova, 2018). We preregistered using the raw fit differences, but verified that strategy assignment remained the same whether we used raw fit differences or relative fit differences.

### Results

#### Prospective memory judgments during training

**Figure 2:**
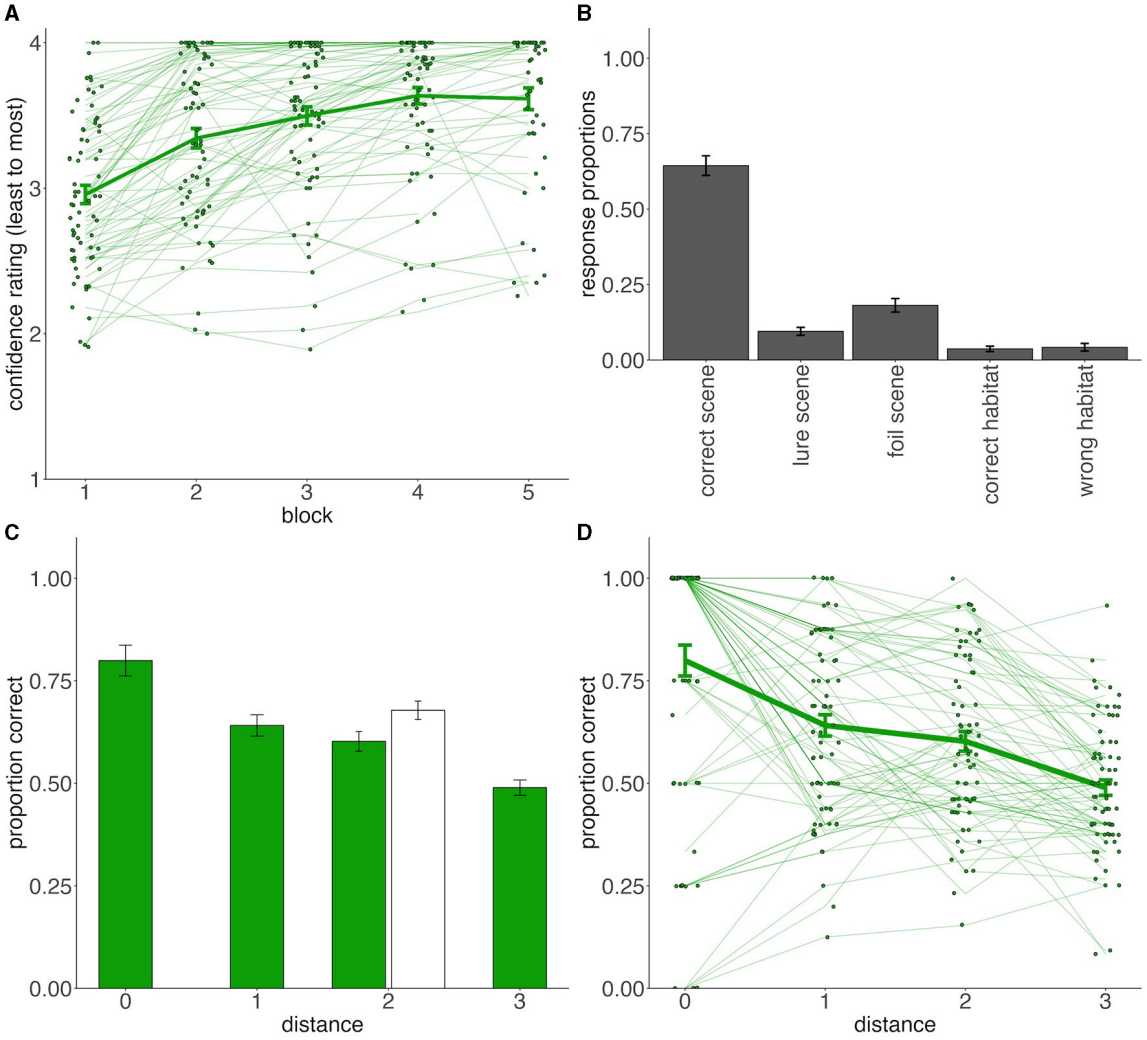
Experiment 1 training and generalization performance. **(A)** Mean confidence ratings for each training block. Thick line indicates the group average ratings, illustrating the increased confidence with training time. Thin lines depict average ratings for each participant. **(B)** Associative memory performance. Bar heights indicate proportion of associative test responses. **(C)** Generalization phase accuracy per each distance from the prototypes. Color indicates stimulus type (white = old; green = new). All training (old) items were at the distance 2 from their respective prototypes. **(D)** Generalization phase accuracy per each distance from the prototypes, including visualization of individual participants. The thick green line indicates the group average [same as **(C)**]. Dots and connecting thin lines represent individual subjects. In all panels, error bars represent standard errors of the mean.

We found a main effect of block [F_(4, 192)_ = 48.2, η^2^ = 0.21, *p* < 0.001], driven by participants increasing confidence in their ability to remember the animal-scene associations over time (Figure 2A). This effect indicates that participants paid attention to the task.

#### Associative memory

We assessed how often participants correctly recalled each training exemplar's paired background scene. Overall, people chose the correct scene most often (M = 64%, SD = 27%) indicating that participants successfully bound training exemplars to their unique background scenes. The full distribution of responses to all options is visualized in Figure 2B and suggests that when participants did not remember the specific correct scene association, they did not seem to remember the general category either. For example, among the three incorrect scenes, they would pick the single lure scene (9.5% of trials) just as often as one of the two foil scenes from the wrong category (18.1% for the two foil scenes = approximately 9% for each foil; Figure 2B). Moreover, the general habitat labels were not used frequently but also did not show any evidence that participants would favor the correct habitat over wrong habitat {t_(70)_ = −0.50, 95% CI_difference_ = [-0.03, 0.02], d = 0.06, *p* = 0.616}. Thus, participants seemed to most often encode the specific scene-animal associations and did not seem to be encoding the scenes at the generic category level.

#### Categorization accuracy

The overall average categorization accuracy was 62% (SD = 16%), which was well above chance {t_(70)_ = 6.19, 95% CI = [0.58, 0.66], d = 0.73, *p* < 0.001}. Participants not only remembered the habitat type for the training animals (M = 68%, SD = 19%), but also tended to correctly guess habitats for new animals (M = 60%, SD = 17%). Categorization accuracy split by the distance to the prototype is presented in Figure 2C, D. To test for generalization gradients among the new test exemplars, we used a repeated measures ANOVA with distance from the category prototype as a predictor and average categorization accuracy as an outcome. We found a main effect of distance (0–3) [F_(3, 210)_ = 39.6, η^2^ = 0.189, *p* < 0.001], with accuracy decreasing with the distance from prototypes (Figure 2D).

As all old (training) exemplars were at distance 2 from the prototypes, we also compared categorization for old vs. new distance 2 exemplars. Consistent with prior work, we found higher categorization accuracy for old than new exemplars at distance 2 from prototypes {t_(70)_ = 3.02, 95% CI_difference_ = [0.03, 0.13], d = 0.39, *p* = 0.004; Figure 2B}. Nevertheless, participants were still more successful categorizing the never-seen prototypes than the directly studied training exemplars {t_(70)_ = 4.52, 95% CI = [0.07, 0.17], d = 0.46, *p* < 0.001}. Thus, the paired-associate learning task produced a pattern of categorization performance similar to a traditional supervised training task, including the categorization advantage for prototypes over training items themselves.

#### Categorization strategies

While a number of sophisticated categorization models exists, prototype and exemplar models represent two points on a continuum of conceptual knowledge from generality to specificity. Thus, we used them here to augment our analysis to determine what kinds of representations people form and utilize for categorization decisions.

**Figure 3:**
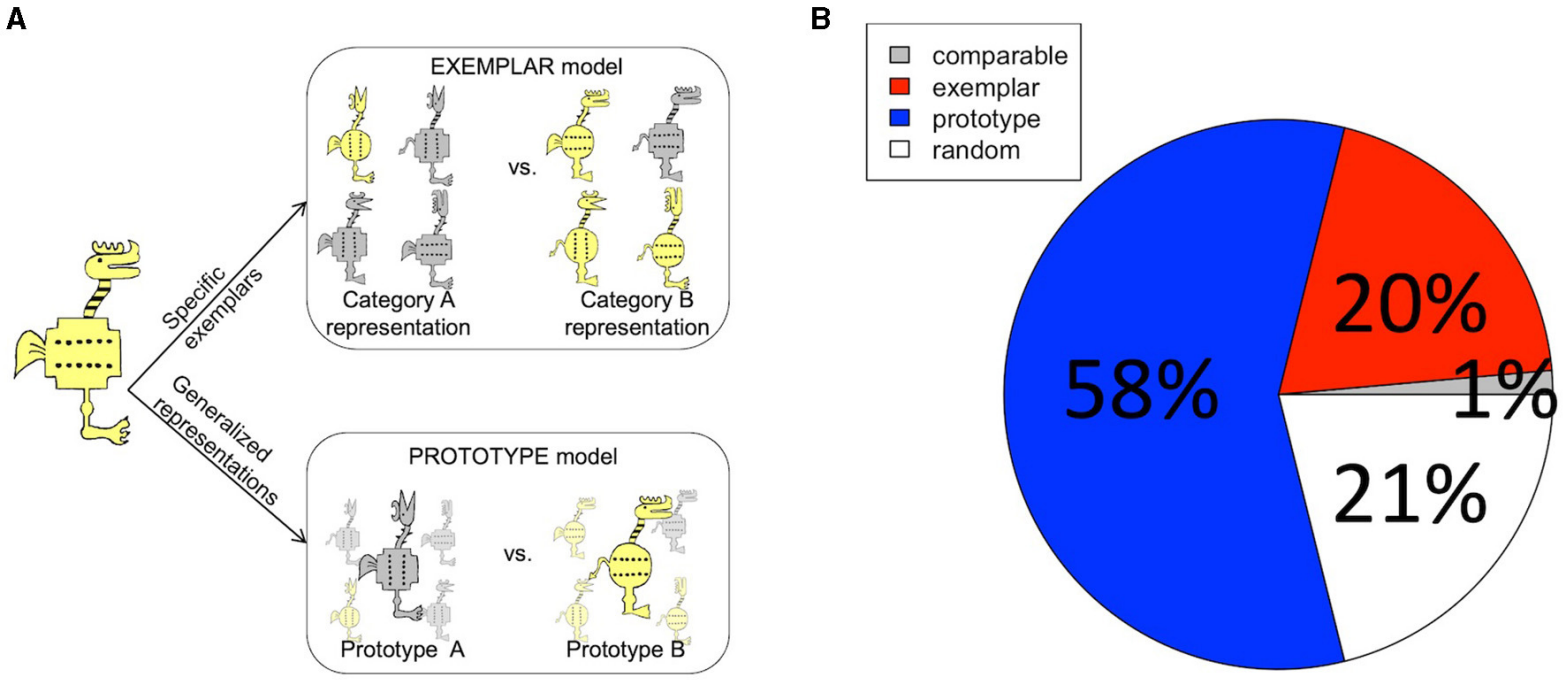
Experiment 1 computational model fitting results. **(A)** Example of the two accounts of generalization that we tested for. Upon seeing a novel stimulus (animal on the left side), one can either compare it to all training exemplars and use the summed similarities within categories to make a response (exemplar model) or compare it to abstracted prototypes to make a response (prototype model). **(B)** The proportion of subjects classified as generalizing according to the prototype strategy, exemplar strategy, having a comparable prototype and exemplar model fit, or having fits not exceeding chance (random).

The conceptual illustration of the prototype and exemplar models is presented in Figure 3A. The proportions of participants that received each label (prototype, exemplar, comparable fit, random) is depicted in Figure 3B. Approximately 58% (41 out of 71) of participants had responses best fit by the prototype model, 20% (14 out of 71) were best fit by the exemplar model, and 1% (1 out of 71) of participants had comparable prototype and exemplar model fits (Figure 3B). The remaining 21% (15 out of 71) of participants had neither prototype nor exemplar model fit reliably above chance and were classified as using a random strategy. Thus, contrary to our prediction, the majority of participants were still best fit by the prototype model, suggesting that they abstracted the central tendency of the category rather than primarily relying on their memory for specific animal-scene associations.

### Discussion

Overall, the results from Experiment 1 provide strong evidence for people's ability to form category knowledge incidentally through association with information that itself has category structure. Notably, participants acquired the category structure even when the task at hand required them to differentiate the specific associated scenes (e.g., all the forests from each other) rather than treating them as equivalent category members. Furthermore, participants seemed to base their categorization judgments on abstract category representations based on the category center (prototypes) rather than the specific associations they acquired for the training stimuli. First, participants showed a typical categorization gradient, and categorized novel prototypes better than the actual training exemplars that they were instructed to remember. Intuitively, generalization gradients are thought to emerge from prototype-based categorization representations but exemplar representations can also produce such a gradient (Jäkel et al., 2007, 2008; Nosofsky and Kruschke, 1992). Because only a formal comparison can determine whether the model predictions provide a good quantitative rather than just qualitative fit (Smith, 2002), we employed formal model fitting to directly estimate each participant's generalization strategy from their pattern of responses. Contrary to our prediction, we found that a majority of participants' responses were fit best by the prototype model. The dominance of prototype strategies in this task is consistent with a previous study (Bowman and Zeithamova, 2018) that used identical stimuli and category structures but employed traditional feedback-based classification training. It was however contrary to our prediction that generalization after the paired-associate learning would be based primarily on exemplar representations. While both the current study and the prior study with the same stimuli found prototype dominance, the specific proportions were numerically shifted toward exemplar representations in our data; we found 58% participants best fit by the prototype strategy compared to 73% in Bowman and Zeithamova (2018) and 20% participants best fit by exemplar model, compared to just 10% in Bowman and Zeithamova (2018). Unsure of whether these are meaningful differences driven by the paired-associate learning task in comparison to the traditional feedback-based classification training, we preregistered and designed Experiment 2. Our goal was to replicate Experiment 1 findings as well as directly compare paired associate learning with feedback-based classification training using a between-subjects design.

## Experiment 2

### Method

#### Participants

Our pre-registered sample size was *N* = 110 (55 for each group; https://osf.io/dzr6v). This was based on a power analysis for a Chi squared test of independence, to provide 80% power to detect medium effects (Effect size w = 0.3) for the difference in strategies between the two groups, as determined in G-Power (Faul et al., 2007, 2009), with a cushion for potential exclusion. The final sample size was larger than our pre-registered sample size as we were posting timeslots in batches every two weeks, as planned in our pre-registration.

Healthy young undergraduate students (*n* = 135; 95 females) were recruited from the University of Oregon via the university's SONA research system and received a course credit for their participation. Participants ages ranged from 18 to 25 (M = 19) years. All participants provided written informed consent and experimental procedures were approved by Research Compliance Services at the University of Oregon.

#### Materials

##### Stimuli

**Figure 4:**
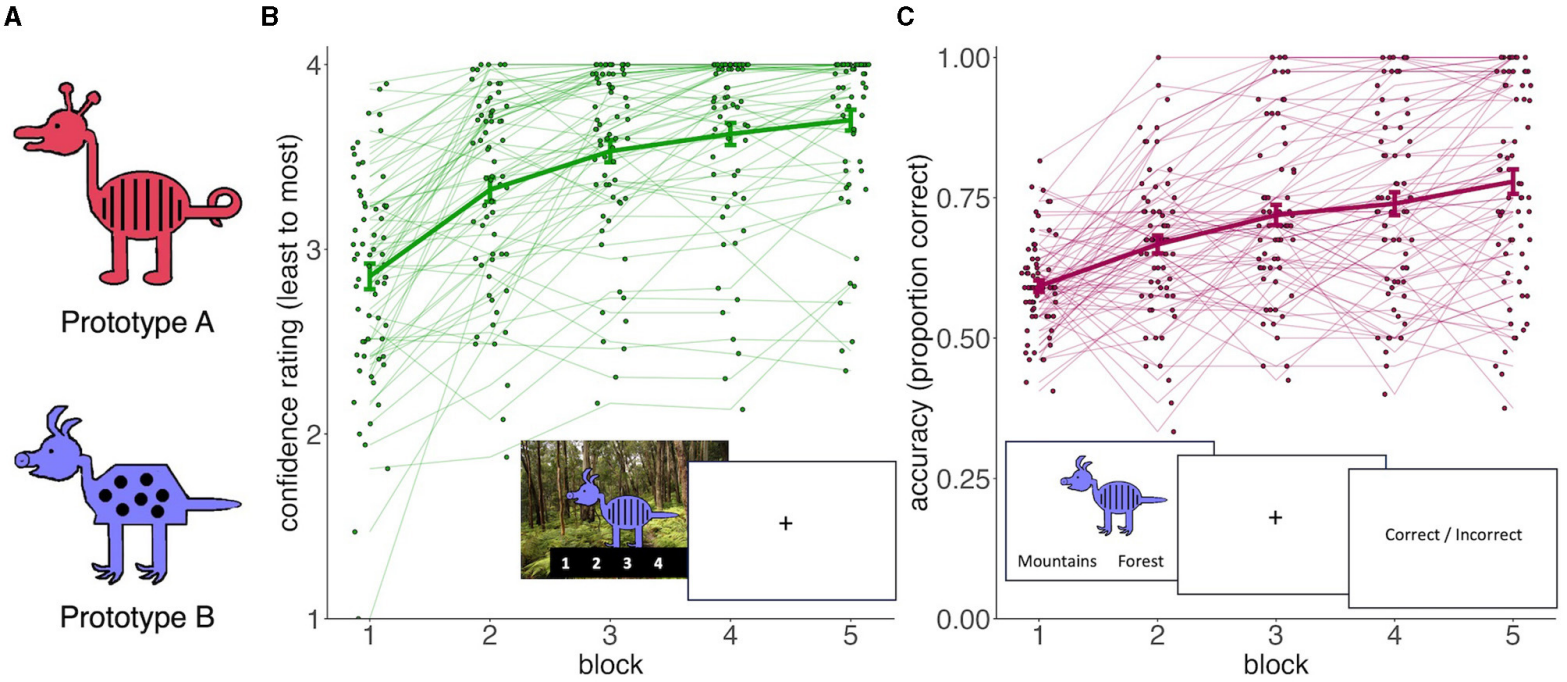
Experiment 2 stimuli and training performance. **(A)** Two example prototypes used in Experiment 2, illustrating the 8 binary features on which the cartoon animals can differ. **(B)** Training responses for paired-associates learning group (confidence ratings). Inset shows an example training trial in the paired-associates learning condition, consisting on observing animal-scene association and rating how likely the association will be remembered on a future test. **(C)** Training performance for feedback-based classification training group (proportion correct). Inset shows an example supervised classification training trial, consisting of categorizing an animal into one of two categories and receiving feedback. In **(B, C)**, thick lines represent group averages and the thin lines and dots represent individual subjects (dots jittered to avoid overlap). Error bars are for standard errors of the mean.

The logical structure of the stimuli was identical to Experiment 1 but the appearance and specific features were different to further test replicability of Experiment 1 with new stimuli. The cartoon animals in Experiment 2 varied along the following 8 binary feature dimensions: color (purple/red), neck (short/long), tail (straight/curled), foot shape (claws/round), snout (rounded/pig), head (ears/antennae), body shape (angled/round), and design on the body (polka dots/stripes). Two example prototypes are presented in Figure 4A. The complete stimulus set is freely available through the Open Science Framework (http://osf.io/8bph2). The structure of the training set and testing set were identical to Experiment 1.

#### Experimental design

Experiment 2 utilized a between-subjects design, where participants were randomly assigned into a paired-associate learning group (*N* = 67) or a feedback-based classification training group (*N* = 68).

Participants in the paired-associate group completed the exact same tasks (paired-associate learning, associative memory test, and category generalization test) as participants in Experiment 1. As in Experiment 1, the paired-associates learning task required participants to memorize eight unique animal-scene association over 5 blocks of training, with five repetitions of each association per block (200 learning trials in total). Training animals from one category were associated with four different mountain scenes, training animals from the other category were associated with four different forest scenes. To ensure attention and equate response requirement with the feedback-based classification group, participants were asked to indicate on each trial how likely they are to remember each animal-scene association on a subsequent test. The paired-associate learning task was followed by an associative memory task for the 8 animal-scene associations, identical to Experiment 1.

Participants in the feedback-based classification training group were learning about the two categories directly, using traditional supervised category learning task with the same stimuli (no scenes). Prior to training, participants were told they would learn the habitats in which different animals lived. During training, participants viewed individual cartoon animals on a computer screen, with a prompt “Does this guy live in the forest or the mountains?” displayed above the animal and two response options on the left (“Mountains”) and the right (“Forest”) side of the screen. Participants choose one of the two categories by pressing either 1 or 0 on the keyboard. Participants had 5 s to make a decision but the trial advanced if a response was registered sooner. After making a response, participants were given feedback for 3 s as to whether they were correct or incorrect (e.g., “Correct!” or “Incorrect. This one was from the mountains.”). As in the paired-associate learning group, there were five training blocks with five repetitions of each of the 8 training exemplars in each block (200 training trials in total).

The category generalization test was the same as in Experiment 1 and identical for both groups. On each trial, participants saw a cartoon animal and had to guess the habitat (“Mountains” or “Forest”) of that animal. The test was self-paced and no feedback was given.

#### Statistical analysis

Raw data and the code necessary to reproduce the plots for Experiment 2 can be found at https://osf.io/dhmks.

##### Training

To test whether participants in the paired-associate learning group maintained attention and participants in the feedback-based classification training group learned over time, we conducted two separate repeated measures ANOVAs using mean confidence ratings (paired-associate learning group) and mean accuracy (feedback-based classification training group) for each participant and training block.

##### Associative memory

We computed associative memory measures for participants in the paired-associate group the same way that we did in Experiment 1.

##### Categorization test accuracy and strategy analysis

For the final category generalization test, we reported the same analyses as in Experiment 1 within each group separately and then compared the groups on each measure. Categorization accuracy was compared using an independent samples *t*-test (overall) and two mixed design ANOVAs (generalization gradient and old/new comparisons). To compare strategies, we used a Chi square test of independence to determine whether the proportions of people classified as using one strategy vs. another differed between groups.

### Results

#### Training data

There was a main effect of block in both the paired-associate learning group [F_(4, 248)_ = 73.9, η^2^ = 0.193, p < 0.001] and feedback-based classification training group [F_(4, 264)_ = 41.2, η^2^ = 0.173, p < 0.001]. Thus, participants trained to remember specific associations became more confident in their ability to remember these associations over time while participants trained to categorize exemplars became more accurate over time (Figures 4B, C). Ergo, both groups were engaged in their respective tasks.

#### Associative memory (paired-associate group only)

**Figure 5:**
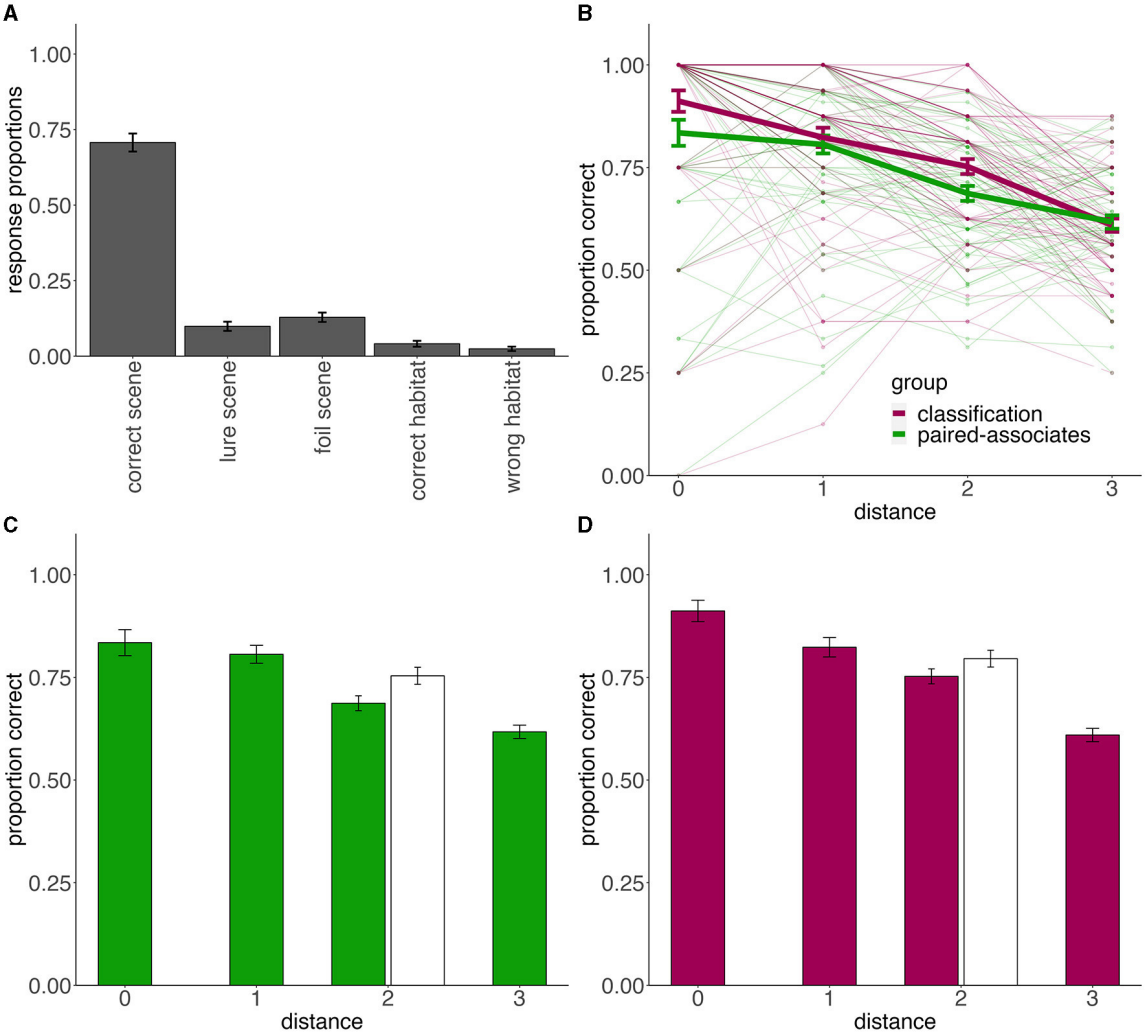
Experiment 2 test performance. **(A)** Associative memory responses for paired-associate group. Bar height indicates the proportion of each type of response. **(B)** Categorization performance for both groups for novel stimuli at each distance to the prototypes. Thick lines represent group averages and thin lines and dots represent individual subject data. **(C)** Categorization performance for just the paired-associates learning group. **(D)** Categorization performance for just the feedback-based classification training group. In **(C, D)**, bar height denotes accuracy at the given distance, averaged across subjects. White bars are old (training) exemplars. In all plots, error bars denote the standard error of the mean.

As in Experiment 1, we assessed how often participants correctly recalled each training exemplar's paired background scene. Overall, people chose the correct scene most often (M = 71%, SD = 24%) indicating that participants successfully bound training exemplars to their unique background scenes. The full distribution of responses to all options is visualized in Figure 5A and, as in Exp. 1, suggests that when participants did not remember the specific correct scene association, they did not seem to remember the general category either. Thus, we replicated the finding from Exp. 1 that participants seemed to most often encode the specific scene-animal associations.

#### Categorization accuracy

During the final category generalization test, participants performed well-above chance in both the paired-associate learning group {M = 72%, SD = 13%, t_(66)_ = 14.272, 95% CI = [0.69, 0.76], d = 1.74, p < 0.001} and feedback-based classification training group {M = 75%, SD = 13%, t_(67)_ = 15.786, 95% CI = [0.72, 0.79], d = 1.91, p < 0.001}. The overall accuracy did not significantly differ between the groups {t_(133)_ = 1.38, 95% CI = [-0.01, 0.08], d = 0.24, p = 0.171}. Thus, categorization during the generalization test was nearly as successful after the paired-associate learning task as after traditional feedback-based classification training. This was quite surprising given that participants in the paired-associate learning group focused on learning specific scene associates of specific animals while participants in the feedback-based classification training group were directly learning to categorize the animals into two categories.

Categorization accuracy for all novel items at each distance from the prototypes is presented in Figure 5B, and with accuracy for old items included in Figures 5C, D. To test for the gradient effect among novel test exemplars and any distance-specific differences between groups, we employed a mixed design ANOVA with distance from the prototype as a within-subject factor and training group (paired-associate learning vs. feedback-based classification training) as a between-subject factor (Figure 5B). We found a main effect of distance [F_(3, 399)_ = 96.05, η^2^ = 0.226, *p* < 0.001], indicating that performance decreased with distance from the prototype, no main effect of group [F_(1, 133)_ = 2.52, η^2^ = 0.008, *p* = 0.115], and a significant group-by-distance interaction [F_(1, 399)_ = 2.98, η^2^ = 0.007, *p* = 0.031]. To follow up on this interaction, we ran *post-hoc* independent samples *t*-tests, comparing group performance at each distance (0–3) from the prototypes. The feedback-based classification training group showed better categorization of distance two items {t_(133)_ = 2.54, 95 % CI = [0.01, 0.12], d = 0.44, *p* = 0.012}, with a similar trend for prototypes {t_(133)_ = 1.88, 95% CI = [0.01, 0.16], d = 0.32, *p* = 0.062}, while distance 1 and distance 3 items were comparable across groups (both |t| < 0.53, d < 0.09, *p* > 0.59). Thus, although we found no reliable differences in the overall accuracy, there seemed to be a small generalization advantage after feedback-based classification training for some of the generalization stimuli.

We next looked at old/new differences among distance 2 items and how they compare between groups. We found a main effect of stimulus type [F_(1, 133)_ = 20.418, η^2^ = 0.028, *p* < 0.001], such that participants categorized training (old) stimuli better than novel ones. We also found a main effect of group [F_(1, 133)_ = 4.73, η^2^ = 0.027, *p* = 0.031], with greater accuracy after feedback-based classification training than paired-associates learning. There was no group-by-old/new interaction [F_(1, 133)_ = 0.94, η^2^ = 0.001, *p* = 0.333], suggesting that the old-advantage was comparable between groups.

#### Categorization strategies

**Figure 6:**
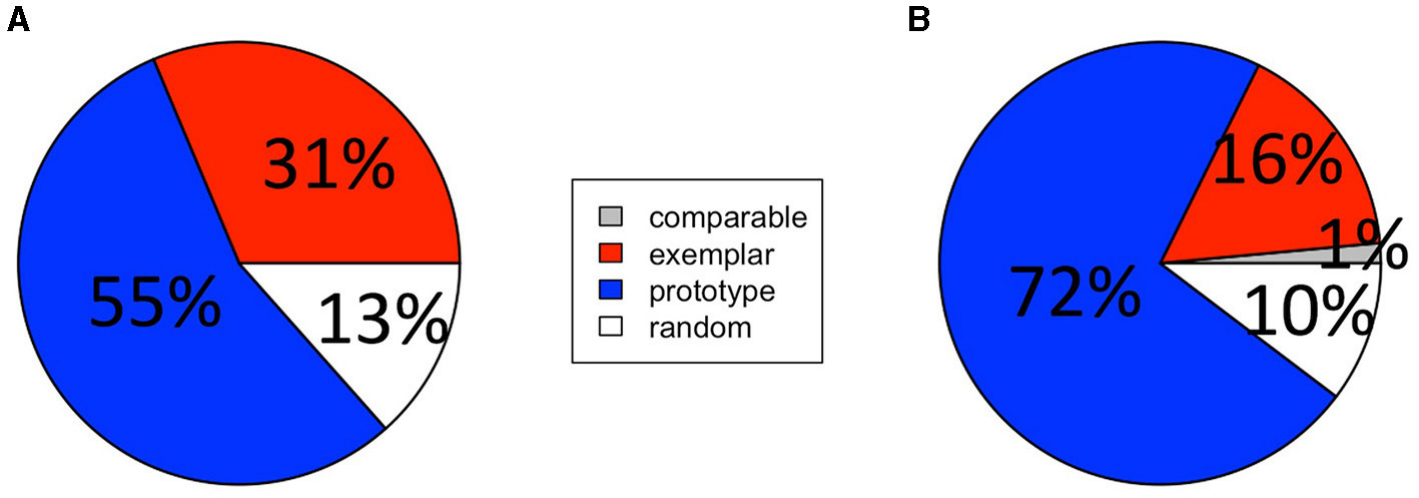
Experiment 2 categorization strategies. The percentage of participants in the **(A)** paired-associate learning group and **(B)** feedback-based classification training group that were assigned each of four possible categorization strategy labels.

The percentage of participants labeled with each strategy label is presented in Figure 6. To compare the proportions of exemplarists and prototypists across groups, we used a Chi-squared test of independence. Only participants that were confidently assigned one or the other strategy were included in the analysis, participants with comparable fits or that used a random strategy were excluded from this analysis. The results showed a trending relationship between generalization strategy and group ($X^{2}$ = 5.73, *p* = 0.057). Thus, while generalization strategies following incidental category acquisition through paired-associates learning were similar to generalization strategies following traditional feedback-based category learning, there was a numerical shift toward exemplar-based strategies.

#### Relationship between specific and general knowledge (Exp. 1 and 2)

We next conducted a (preregistered) exploratory analysis that attempted to better understand the relationship between specific and general knowledge. Many theories suggests that generalization and specificity may trade off, such that formation of generalized memory representations comes at the expense of memory for specific details (McClelland and Goddard, 1996; Shohamy and Wagner, 2008; Varga et al., 2019). Because the paired-associates training group was tested not only on concept generalization but also on their memory for specific animal-scene associations requiring them to distinguish among similar animals and among similar scenes, we utilized the opportunity to explore the potential trade-off at the level of individual differences.

To look for a potential trade off in our data, we first tested whether participants who successfully formed generalized knowledge were less able to remember specific details. We reasoned that if prototypists formed general knowledge while exemplarists formed specific memories, then exemplarists should demonstrate superior associative memory performance.

**Figure 7:**
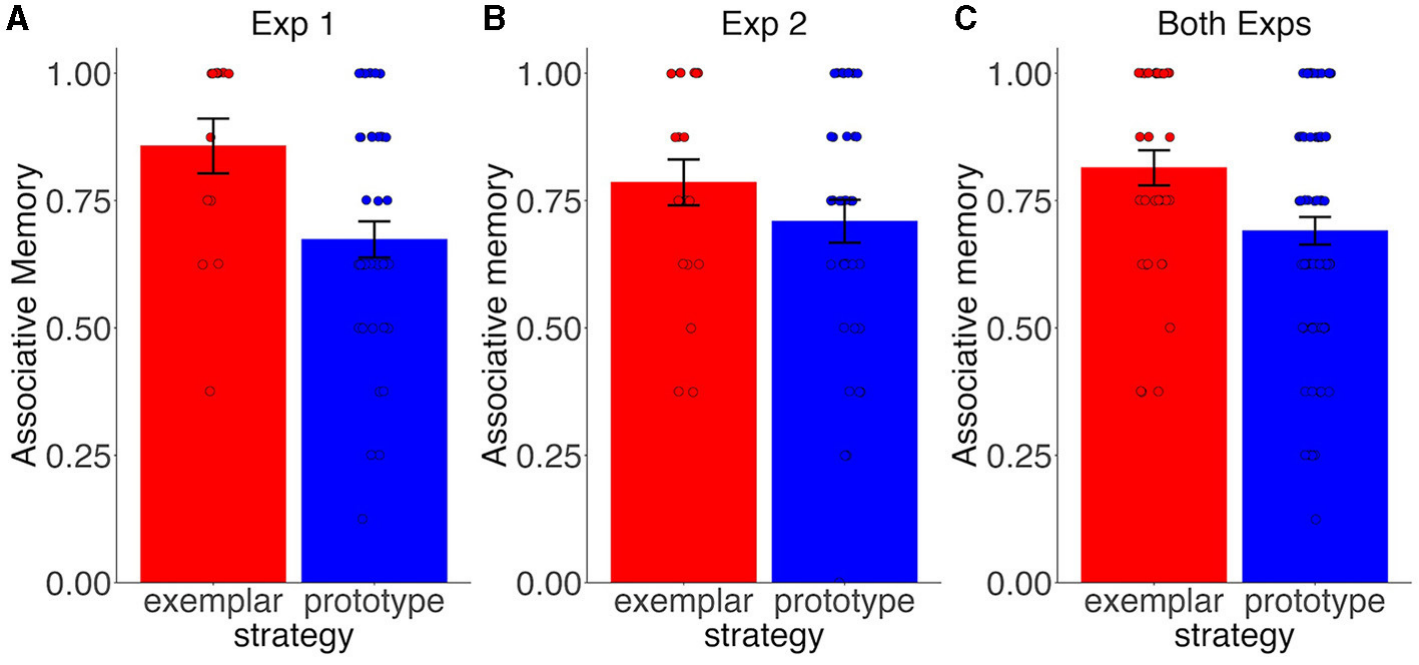
Associative memory and subsequent strategy use. Associative memory performance for prototype and exemplar strategy users in Exp. 1 **(A)**, Exp. 2 **(B)**, and when collapsed across both experiments **(C)**. Error bars are standard errors of the mean. Dots denote individual participants. Colors denote generalization strategy (red = exemplarists; blue = prototypists).

Using a two sample *t*-test comparing associative memory between participants classified as “exemplarists” vs. “prototypists”, we found significantly greater associative memory for exemplarists than prototypists in Exp. 1 [t_(53)_ = 2.68, d = 0.83, p = 0.010; Figure 7A] and numerically greater associative memory for exemplarists than prototypists in Exp. 2 [t_(56)_ = 1.17, d = 0.32, p = 0.248; Figure 7B]. The difference between exemplarists and prototypists was also significant when data were collapsed across experiments [t_(111)_ = 2.65, d = 0.54, p = 0.009; Figure 7C]. Thus, we found some evidence that the formation of generalized category representation, operationalized here as the use of a prototype strategy, came at the expense for memory for specific animal-scene associations.

**Figure 8:**
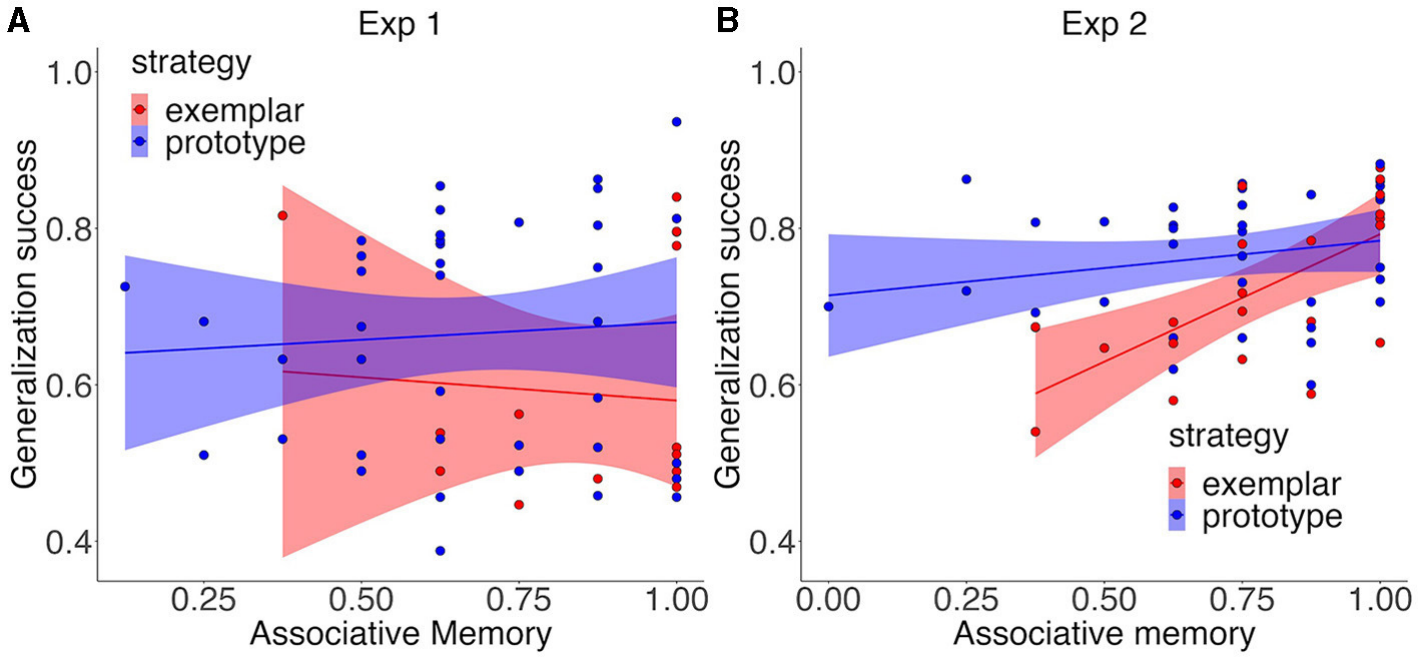
Generalization-associative memory relationship. Lines represent correlations between correct scene responses (y-axis) and generalization accuracy (x-axis) and ribbons denote 95% confidence intervals for Exp. 1 **(A)**, Exp. 2 **(B)**. Dots denote individual participants. Colors denote generalization strategy (red = exemplarists; blue = prototypists).

Next, we were interested whether memory for specific details and generalization success are related in our sample, and whether this relationship differs depending on categorization strategy. While generalization decisions can be based both on specific memories and generalized knowledge, making predictions for the specificity-generalization relationship uncertain, the formal models are helpful in estimating the underlying memory representations underlying categorization behaviors. For example, specificity and generalization may go hand-in-hand instead of trading off when they are both dependent on the same memory representations (Richter et al., 2016). We hypothesized that, if people used an exemplar-based strategy to categorize stimuli, their generalization and associative memory performance should be positively related. Such a relationship may not exist among prototypists as they presumably use different representations for the associative memory and generalization tasks. To test these ideas, we computed across-subjects correlations between associative memory scores and generalization accuracy during the final categorization test, separately among prototypists and exemplarists. The results were inconsistent across experiments. In Exp 1, we found no relationship between associative memory and generalization for either exemplarists (r = −0.08, 95% CI = [−0.59, 0.47], p = 0.782) or prototypists (r = 0.07, 95% CI = [-0.24, 0.37], *p* = 0.670) (Figure 8A). In Exp. 2, we found a strong positive correlation between associative memory and generalization for exemplarists (r = 0.66, 95% CI = [0.32, 0.85], *p* = 0.001) but not for prototypists (r = 0.22, 95% CI = [-0.11, 0.51], p = 0.181) (Figure 8B). Regression analysis directly comparing the prototype and exemplar strategy users confirmed that the relationship between associative memory and generalization was reliably stronger for exemplarists than prototypists (strategy ^*^ associative memory interaction, β = 0.26, *p* = 0.013). Thus, we found partial evidence that the relationship between specific and general knowledge does differ depending on strategy, though the results were inconsistent across experiments.

## General discussion

The complexity of the world allows us to acquire category knowledge in a number of ways. Most work evaluating category knowledge acquisition trains people to learn categories using category response options coupled with explicit feedback, such that people are trained to associate stimuli with category labels through trial-and-error. In the current paper, we were interested in whether the same category knowledge could be acquired incidentally, without explicit instruction, by learning to associate individual category members with other specific stimuli that can be organized into categories based on preexisting knowledge. We found that people did indeed acquire category knowledge despite training to remember specific paired associates, reaching nearly identical generalization performance as those explicitly trained to categorize. Moreover, similarly to those who underwent explicit feedback-based categorization training, paired associate learners showed a prototype strategy dominance during categorization test. Nevertheless, a marginal trend suggested a tendency toward more exemplar-based strategies in people trained to remember specific information. These findings inform our understanding of how memories for experiences are spontaneously organized to serve multiple forms of cognition.

Results from the current study underscore important similarities and differences between incidental and more traditional category learning. Incidental category learning may provide a more naturalistic framework to characterize categorization processes (Broadbent et al., 2018; Gabay et al., 2015, 2023; Roark et al., 2022; Unger and Sloutsky, 2022). For example, a child that hears the word “ball” might already be focusing on its red and spherical properties, and thus, will incidentally learn to associate the category *ball* with red and sphere features. Indeed, a number of studies have implicated incidental category structure in learning relational information in children (Gentner and Namy, 2006; Vasilyeva et al., 2018). In the current study, we administered a novel category learning paradigm that resembles incidental learning, in that participants never received either explicit instruction to categorize stimuli or corrective feedback during training. However, unlike the majority of prior incidental categorization studies, the categories were not uniquely dictated by a similarity structure among training exemplars. Despite that, we found evidence for robust incidental concept learning after learning to remember specific associations. While the categorization performance was slightly better in the feedback-based classification training group for some of the stimuli, including the prototypes, the overall accuracy was comparable. Participants trained to remember specific associations were able to generalize incidentally-aligned category structure to novel stimuli, and showed similar generalization gradients as typically found in traditional category training studies, such as higher categorization success for prototypes than the stimuli they were trained on. As such, this study contributes to our understanding of concept learning under more naturalistic demands and indicates that many aspects of category learning are robust with respect to how the categories are learned.

Prior work on incidental learning has provided evidence that the learning systems and cognitive mechanisms engaged by traditional, feedback-based learning may be distinct from those employed during incidental category learning (Lim et al., 2019; Tricomi et al., 2006). Furthermore, research on episodic memory has shown how task goals and other conditions may bias participants to encode related information into separated memories vs. integration representations (Chanales et al., 2019, 2021; Schlichting et al., 2014, 2015; Schlichting and Preston, 2015; Zeithamova and Preston, 2017). Here, we hypothesized there would be a difference in generalization strategy between feedback-based classification training group and paired-associate learning group. Specifically, we used prototype and exemplar models of categorization, both of which can provide good fits to behavior (Bowman et al., 2022; Bowman and Zeithamova, 2020, 2023; Heit, 1992; Hintzman, 1984; Nosofsky, 1986; Nosofsky et al., 1994; Posner and Keele, 1968) and neural activity (Bowman et al., 2020; Bowman and Zeithamova, 2018; Mack et al., 2013). We predicted an increase in reliance on exemplars after paired-associate learning emphasizing memory for individual stimuli, but the results were mixed in that regard. Although we found a marginally significant shift toward exemplar strategies after paired-associate learning, more than half of the participants were still fit best by the prototype model. As a second approach to comparing the role of specific memories in generalization success, we evaluated the degree of “old-advantage”, or better categorization performance for old items than new items at the same distance from the prototypes, which can indicate a role for exemplar memorization in the overall categorization success. Old-advantage in both experiments was reliably above zero, but comparable between paired-associate learning group and feedback-based classification training group. Thus, the role of exemplar memorization, as indicated by the old-advantage analysis, did not seem to increase after the paired-associate learning. One possible reason for a lack of increased exemplar memorization following paired-associate learning is that the common scenes facilitated more contextual-based memory than memorization of associations (Hayes et al., 2007). That is, perhaps visual context (i.e., mountains or forest) facilitated retrieval of central tendencies within each context.

Exploratory analyses focused on the relationship between specificity and generalization. First, we hypothesized that given their reliance on specific memories, exemplarists may have better memory for animal-scene associations than those relying on prototypes. The data were relatively well aligned with this prediction. This may indicate that there is a trade-off between specificity and generalization, such that the formation of a prototype comes at the expense of memories for specific associations. Alternatively, those with superior associative memory may be more likely to adopt an exemplar strategy. Second, we hypothesized that individual differences in associative memory may track generalization success in those relying on an exemplar strategy, given that both tasks are presumably supported by the same representations. In contrast, such a relationship may be less pronounced in those using a prototype strategy, as they presumably use distinct representations for the two tasks. The results of Experiment 2 aligned with this prediction: we observed a strong positive correlation between associative memory and generalization success in exemplarists, which is predicted by single-system theories assuming that the same type of memory representations supports both specific and generalized judgments (Zeithamova and Bowman, 2020). However, this correlation was only observed for Exp. 2 and Exp. 1, meaning the results are inconclusive. The relationship between associative memory and generalization success in those relying on prototypes was not significant in either experiment, which may reflect the use of distinct representations across the two tasks. However, each experiment was underpowered with respect to individual differences analyses, preventing strong conclusions from these null findings. Future studies may revisit this question in a larger sample, as within-subject correlations can contribute to our understanding of the shared and unique processes across different cognitive measures.

The grouping of participants into exemplar- and prototype-based strategy users assumes that people form representations of either prototypes or exemplars. Decades of debate have reinforced this dichotomy, yet, more recently, it has been theorized that prototypes and exemplars form in parallel in the brain (Bowman et al., 2020; Zeithamova and Bowman, 2020). Forming representations at multiple levels of specificity can promote flexibility in future decision-making, as it is not always clear how current experience will become relevant. Intriguingly, the hippocampal axis has been posited to track multiple scales of information content (Brunec et al., 2018; Maurer and Nadel, 2021), consistent with a gradient of place cell receptive field sizes (Strange et al., 2014). Indeed, neuroimaging studies have found prototype representations specifically in the anterior portion of the hippocampus (Bowman et al., 2020; Bowman and Zeithamova, 2018). Functional connectivity along the hippocampal axis also aligns with the specificity-generalization continuum (Frank et al., 2019) and Guo and Yang (2023) show that the ventromedial prefrontal cortex is more strongly connected with the posterior hippocampus when retrieving specific associations but more strongly connected with the anterior hippocampus when retrieving schemas. While category representations can be found elsewhere in the brain (Mansouri et al., 2020), it is apparent that, across both tasks (Blank and Bayer, 2022; Mack et al., 2013) and methodologies (Sherman et al., 2023), specific and general representations of experience may form in parallel.

The current findings are broadly consistent with the notion that generalized representations may form in parallel with specific ones during learning to be flexibly utilized during subsequent decision-making depending on task goals. While we hypothesized that people trained to remember specific information would increasingly use an exemplar-based strategy, we instead found reliance on prototypes in the majority of participants. Importantly, because the associative test required differentiation among similar animals and background scenes from the same category, it could not be based on just general knowledge about scene categories. Thus, individuals appeared to both encode specific information and construct general representations, and were able to use either type of memory in a flexible, task-dependent manner. Prototype strategy may dominate categorization task in most participants because it is cognitively more efficient. Nevertheless, the increased attention to differentiating details of individual exemplars elicited by the paired-associate learning task may result in somewhat greater exemplar strategy utilization than observed after traditional supervised training.

### Conclusions

In the current study, we have demonstrated that people trained to remember specific associations can incidentally acquire knowledge of category structures revealed by association with other material that itself is structured. Furthermore, generalization of such knowledge to novel stimuli relied on similar representations as when acquired during traditional feedback-based classification training. When considered in tandem with previous literature, these results suggest that people may encode experiences at multiple levels of specificity, maintaining specific details while spontaneously organizing related memories to form more generalized knowledge. This may allow them to flexibly switch among representations, focusing on differentiating details of specific memories vs. generalized knowledge, in response to task demands.

## Data Availability

The datasets presented in this study can be found in online repositories. The names of the repository/repositories and accession number(s) can be found below: https://osf.io/dhmks/.
